# Variation in *RARG* increases susceptibility to doxorubicin-induced cardiotoxicity in patient specific induced pluripotent stem cell-derived cardiomyocytes

**DOI:** 10.1038/s41598-020-65979-x

**Published:** 2020-06-25

**Authors:** Effimia Christidi, Haojun Huang, Sanam Shafaattalab, Agnes Maillet, Eric Lin, Kate Huang, Zachary Laksman, Margot K. Davis, Glen F. Tibbits, Liam R. Brunham

**Affiliations:** 1 Centre for Heart Lung Innovation, Department of Medicine, University of British Columbia, Vancouver, Canada; 2 Molecular Cardiac Physiology Group, Department of Biomedical Physiology and Kinesiology, Simon Fraser University, Burnaby, Canada; 3 Astellas Pharma Europe B.V., Leiden, Netherlands; 4 Heart Rhythm Services, Division of Cardiology, Department of Medicine, University of British Columbia, Vancouver, Canada; 5 Department of Medicine, University of British Columbia, Vancouver, British Columbia Canada; 6 Department of Cardiovascular Science, British Columbia Children’s Hospital, Vancouver, Canada; 7 Department of Medical Genetics, University of British Columbia, Vancouver, British Columbia Canada

**Keywords:** Induced pluripotent stem cells, Cardiovascular genetics

## Abstract

Doxorubicin is a potent anticancer drug used to treat a variety of cancer types. However, its use is limited by doxorubicin-induced cardiotoxicity (DIC). A missense variant in the *RARG* gene (S427L; rs2229774) has been implicated in susceptibility to DIC in a genome wide association study. The goal of this study was to investigate the functional role of this *RARG* variant in DIC. We used induced pluripotent stem cell derived cardiomyocytes (iPSC-CMs) from patients treated with doxorubicin. iPSC-CMs from individuals who experienced DIC (cases) showed significantly greater sensitivity to doxorubicin compared to iPSC-CMs from doxorubicin-treated individuals who did not develop DIC (controls) in cell viability and optical mapping experiments. Using CRISPR/Cas9, we generated isogenic cell lines that differed only at the *RARG* locus. Genetic correction of *RARG*-S427L to wild type resulted in reduced doxorubicin-induced double stranded DNA breaks, reactive oxygen species production, and cell death. Conversely, introduction of *RARG*-S427L increased susceptibility to doxorubicin. Finally, genetic disruption of the *RARG* gene resulted in protection from cell death due to doxorubicin treatment. Our findings suggest that the presence of *RARG-*S427L increases sensitivity to DIC, establishing a direct, causal role for this variant in DIC.

## Introduction

Doxorubicin is an anthracycline chemotherapeutic drug used in the treatment of solid organ and hematological malignancies for both adult and pediatric patients^1^. Despite its effectiveness as an anti-cancer agent, the use of doxorubicin is limited by cardiotoxicity which affects up to one quarter of patients^2^. Doxorubicin-induced cardiotoxicity (DIC) leads to cardiomyopathy with systolic dysfunction, arrhythmia, congestive heart failure, and in some cases death^1,3^. Despite extensive investigation, the mechanisms of DIC remain incompletely understood, and we lack the ability to predict this adverse drug reaction in individual patients.

Genetic association studies have identified several genetic variants that are associated with DIC^4^. However, lack of replication of these findings and the absence of functional validation has precluded their implementation as clinical tests. Among the variants with the strongest genetic evidence is a non-synonymous coding variant (rs2229774, S427L) in the retinoic acid receptor gamma gene, *RARG*, which was identified in a genome-wide association study (GWAS) of DIC (odds ratio = 4.7, p = 5.9 10^−8^)^5^. However, the directionality of this effect is controversial^6^, and genetic association studies cannot prove a causal relationship between a genetic locus and the phenotype of interest. As a consequence, direct functional assessment of the role of this variant in DIC is essential.

Human induced pluripotent stem cell-derived cardiomyocytes (iPSC-CMs) have emerged as a physiologically-relevant model system for studying DIC^7,8^, but to date the impact of specific genetic variants on DIC in this model has not been assessed. The availability of precise genome editing tools, such as CRISPR/Cas9, provides a powerful platform to assess causal relationships between genetic variants and disease processes. iPSC-CMs possess many advantages for studying drug toxicity including their potential for high throughput applications and a higher degree of homology with human cardiomyocytes, compared to animal models^9,10^. In addition, patient derived iPSC-CMs are genetically identical to the patient from whom they were derived, which allows for a direct comparison between the patient’s clinical response and *in vitro* phenotype. The goal of this study was to investigate the functional impact of *RARG-*S427L on DIC in isogenic, patient-derived iPSC-CMs.

## Results

### Increased sensitivity to doxorubicin in patient specific induced pluripotent stem cell-derived cardiomyocytes from individuals who experienced doxorubicin-induced cardiotoxicity

**Figure 1:**
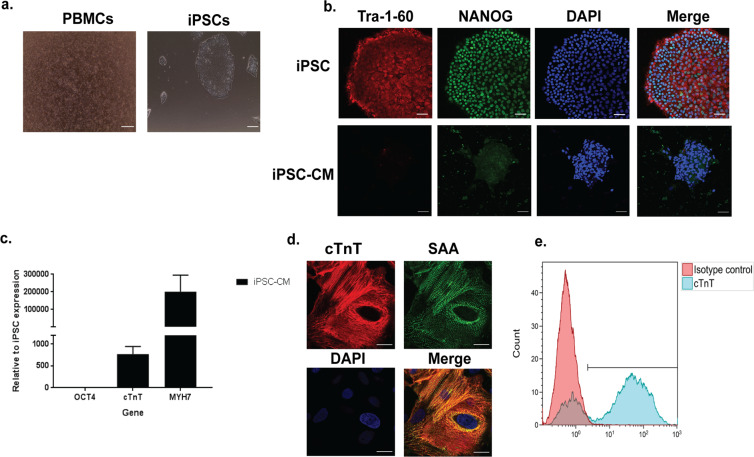
Generation of patient specific induced pluripotent stem cell-derived cardiomyocytes. (**a**) Representative photomicrographs of peripheral blood mononuclear cells and induced pluripotent stem cell (iPSC) colonies. (**b**) Confocal microscopy images showing presence of Tra1–60, NANOG and DAPI, scale bar = 50μm. (**c**) Cardiac Troponin T and Myosin heavy chain 7 (cTnT, MYH7) mRNA expression in iPSC-derived cardiomyocytes (CM), n = 10. Data are shown as mean ± s.e.m. (**d**) Confocal microscopy images showing the presence of cTnT and Sarcomeric α-actinin (SAA), scale bar = 20μm. (**e**) Flow cytometry analysis of cTnT expression in iPSC-derived cardiomyocytes.

We recruited patients who had been treated with an anthracycline based chemotherapy regimen and either developed DIC (cases) or did not (controls) (see Supplementary Table S1 for enrolment criteria). The characteristics of the DIC cases and controls, including genotype for *RARG-*S427L, are shown in Table 1. The mean equivalent doxorubicin dose for cases was 342.5 mg/m^2^ vs. 306.7 mg/m^2^ for controls, (p = 0.6). We isolated peripheral blood mononuclear cells from each patient and performed reprogramming to iPSCs (Fig. 1a). iPSCs displayed robust expression of pluripotency markers (Fig. 1b and Supplementary Fig. S1a). Karyotype analysis showed normal karyotypes in all cell lines with the exception of two passages of one cell line (DIC-023, Supplementary Fig. S2a,b),which showed a possible amplification of the minimal critical chromosomal region 12p, an abnormality often seen in iPSCs^11,12^. Other passages of this clone displayed normal karyotype, as did genome edited cell lines derived from this clone. Trilineage differentiation to the three germ layers confirmed pluripotency of these cells (Supplementary Fig. S1b–d). We performed directed differentiation of the iPSCs to CMs, which expressed typical cardiomyocyte markers such as cardiac troponin T (cTnT, 75% cTnT positive cells), Sarcomeric α-actinin (SAA) and Myosin heavy chain 7 (MYH7) (Fig. 1c–e and Supplementary Fig. S3, S4).

**Table 1:** Characteristics of cases and controls.

Cases	Type of Cancer	Sex	Age at enrolment (years)	Age at treatment (years)	Cumulative Anthracycline Dose (mg/m^2^)	Post treatment Left Ventricular Ejection Fraction (LVEF)	RARG genotype
DIC-001	Breast	F	70	56	300 (DOX)	20%	WT/WT
DIC-002	Sarcoma	F	52	47	450 (DOX)	36%	WT/WT
DIC-003*	Breast	F	87	68	320 (DOX)	36%	WT/S427L
DIC-008	Breast	F	67	48	300 (DOX)	20%	WT/WT
**Controls**							
DIC-014**	Sarcoma	F	32	15	420 (DOX)	60%	WT/WT
DIC-021	Lymphoma	F	27	11	180 (DAUN)	60%	WT/S427L
DIC-023*	Breast	F	65	51	320 (DOX)	55%	WT/WT

*Used for genome editing experiments.

**Used for knock out experiments.

DOX: doxorubicin, DAUN: daunorubicin (DOX:DAUN 1:1 equivalence ratio used by clinical groups for assessment of cardiotoxicity^13–17^).

**Figure 2:**
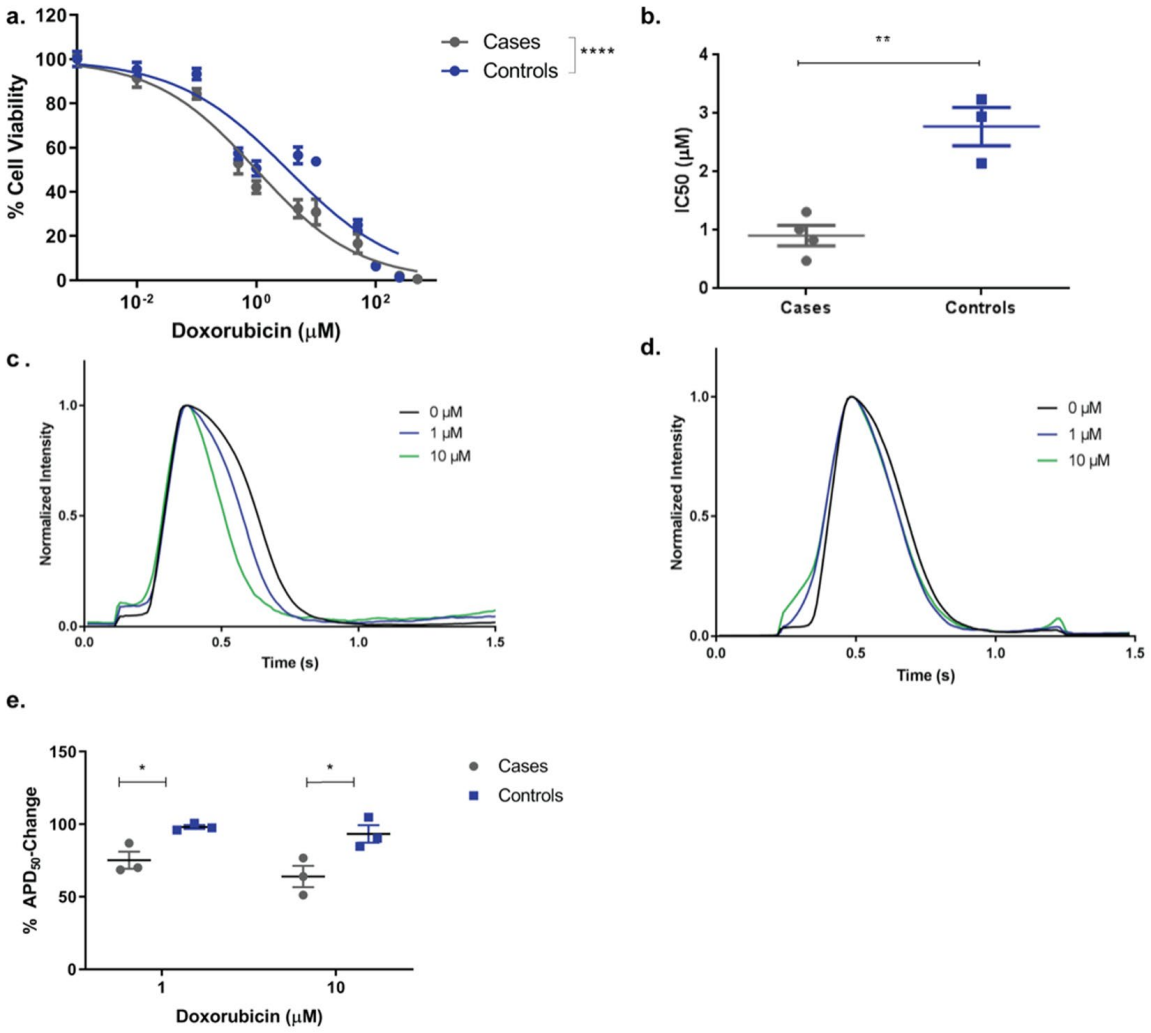
Modelling sensitivity to doxorubicin in patient specific induced pluripotent stem cell-derived cardiomyocytes from cases and controls. (**a**) Cell viability and (**b**) IC_50_ of induced pluripotent stem cell-derived cardiomyocytes (iPSC-CMs) from cases and controls using Cell Titer-Glo after 24 h of doxorubicin treatment. Graphs show pooled data from n** =** 4 cases and n** =** 3 controls, each patient cell line was assessed three independent times, in technical triplicates. (**c**,**d**) Representative optical mapping experiments showing the action potential of one case (**c**) and one control (**d**) cell line in response to increasing doses of doxorubicin, upon 20 mins of doxorubicin incubation. (**e**) Change of action potential duration at 50% of repolarization 1 μΜ and 10 μΜ doxorubicin n** =** 3 cases, n** =** 3 controls, relative to 0μΜ doxorubicin. Data points represent independent, biological replicates of pooled data of cases and controls measured in multiple differentiation batches of one cell line per patient. Data are shown as mean ± s.e.m. *p** <** 0.05, **p** <** 0.01, ****p** <** 0.0001, t-test.

We compared the *in vitro* sensitivity to doxorubicin of iPSC-CMs from DIC cases and controls. iPSC-CMs from DIC cases displayed significantly greater susceptibility to doxorubicin-induced cell death compared to controls (Fig. 2a,b cases: IC_50_ = 0.91 ± 0.35μΜ, pooled data from n = 4 patients, controls: IC_50_ = 2.77 ± 0.57μΜ, n = 3 patients, p = 0.003). This finding confirms that the clinical susceptibility to DIC is mirrored by increased sensitivity to the cardiotoxic effects of the drug *in vitro*.

Doxorubicin causes a variety of electrophysiological changes acutely during its administration including nonspecific ST and T-wave changes, decreased QRS voltage, prolongation of the QT interval and ventricular premature beats^3,18,19^. Previous research in experimental models has shown that doxorubicin can cause shortening or prolongation of the action potential duration (APD)^20–22^. We investigated whether iPSC-CMs from cases are more sensitive to the electrophysiological effects of doxorubicin than controls, by assessing the impact of doxorubicin on voltage transients using optical mapping^23,24^. We observed that doxorubicin caused shortening of the APD in a dose-dependent manner, and that this effect was significantly more pronounced in cases than controls (Fig. 2c,d,e, %change of APD_50_ with 1 μM doxorubicin relative to 0 μM: cases = 75.1 ± 10.1%, controls = 98.1 ± 2.3%, n = 3 patients each, p = 0.04).

### Effect of *RARG*-S427L on doxorubicin induced cardiotoxicity

**Figure 3:**
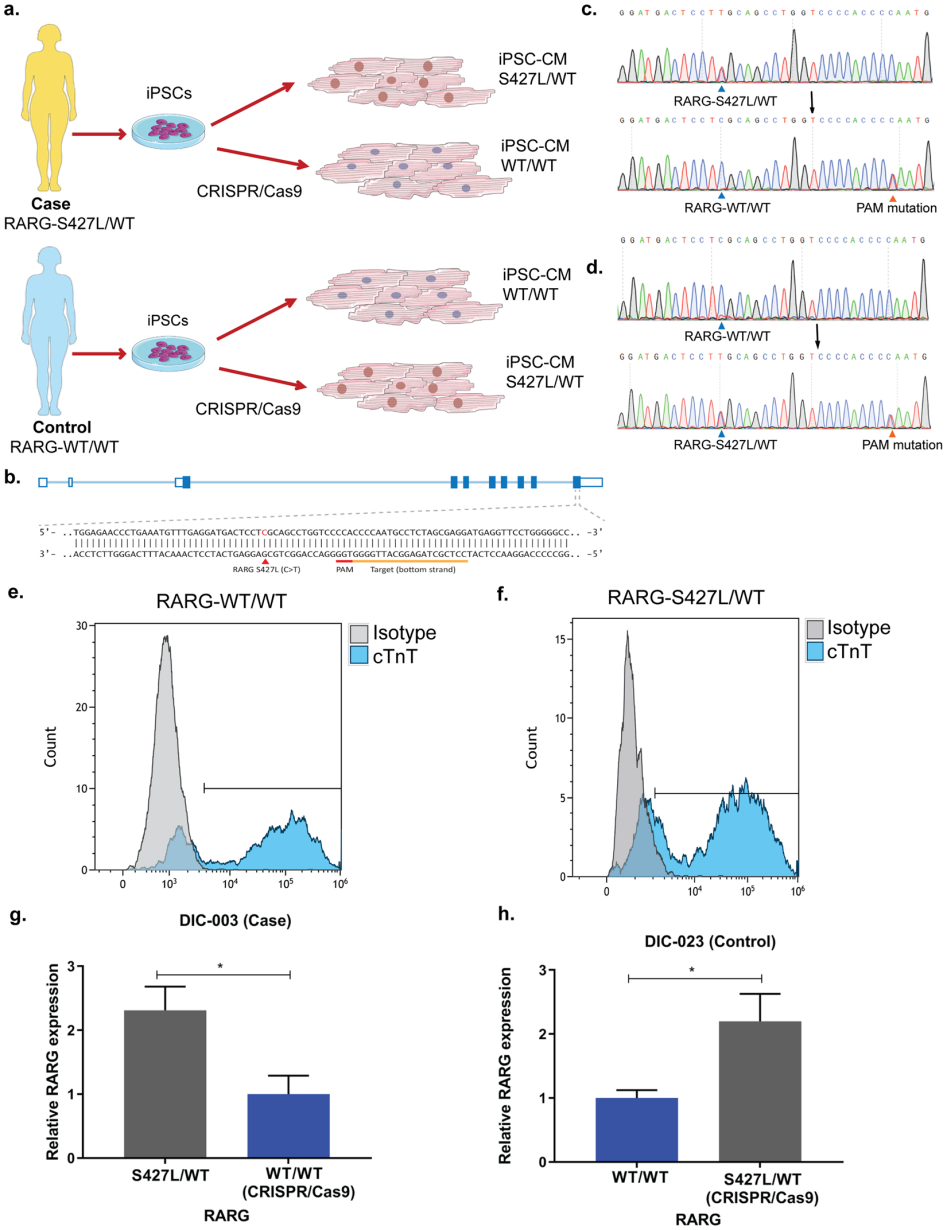
Genome editing experimental approach and comparison of edited and unedited isogenic cell lines. (**a**) Experimental approach of CRISPR/Cas9 experiments, correction of *RARG*-S427L/WT in a case (DIC-003) patient cell line and insertion of *RARG*-S427L in a control (DIC-023) patient cell line. Images have been acquired from Servier Medical Art by Servier licensed under a Creative Commons Attribution 3.0 Unported License. (**b**) Schematic overview of *RARG* locus and CRISPR/Cas9 genome editing approach. (**c**) Sanger sequencing results showing correction of *RARG*-S427L/WT to WT/WT (DIC-003). (**d**) Sanger sequencing results showing introduction of *RARG*-S427L/WT to a control WT/WT patient cell line (DIC-023). Flow cytometry results of cTnT expression in (**e**) RARG-WT/WT and in (**f**) its isogenic genome edited S427L/WT cell line. (**g**) RT-qPCR results of *RARG* expression, in the heterozygous case (DIC-003) and the isogenic corrected WT/WT cell line (**h**) RT-qPCR results of *RARG* expression in the homozygous control (DIC-023) cell line (WT/WT) and the isogenic edited S427L/WT cell line. Data are shown as mean ± s.e.m., n = 3, representing biological, independent replicates *p < 0.05, t-test.

We hypothesized that part of the increased susceptibility to DIC among cases may be mediated by the presence of the *RARG*-S427L variant. To test this hypothesis, we took a two-step approach. First, we performed genome editing to correct this variant to the wild type sequence in a case that was heterozygous for S427L. Secondly, we used a control patient cell line that was homozygous wild type at *RARG* (WT/WT) and introduced the variant (S427L/WT) (Fig. 3a,b, Table 1). Sequencing confirmed successful editing of the two cell lines (Fig. 3c,d) while sequencing of predicted off-target sites did not detect evidence of off-target genome editing (Supplementary Fig. S5a–c). In order to assess that pluripotency and differentiation efficiency of iPSCs was not affected by the genome editing process or the presence of the variant, we performed staining for NANOG and Tra 1-60 (Supplementary Fig. S1a). Both markers were robustly expressed in both genome-edited and unedited iPSCs. In addition, genome-edited and unedited iPSC-CMs displayed similar cTnT expression assessed by flow cytometry (Fig. 3e,f 76% cTnT positive cells in *RARG*-WT/WT and 74% cTnT positive cells in *RARG*-S427L/WT) and by qPCR (Supplementary Fig. S6a,b), suggesting that differentiation efficiency was not altered. *RARG* mRNA expression was significantly higher in cell lines with the *RARG*-S427L variant (Fig. 3g, p = 0.03 and 3h p = 0.03).

**Figure 4:**
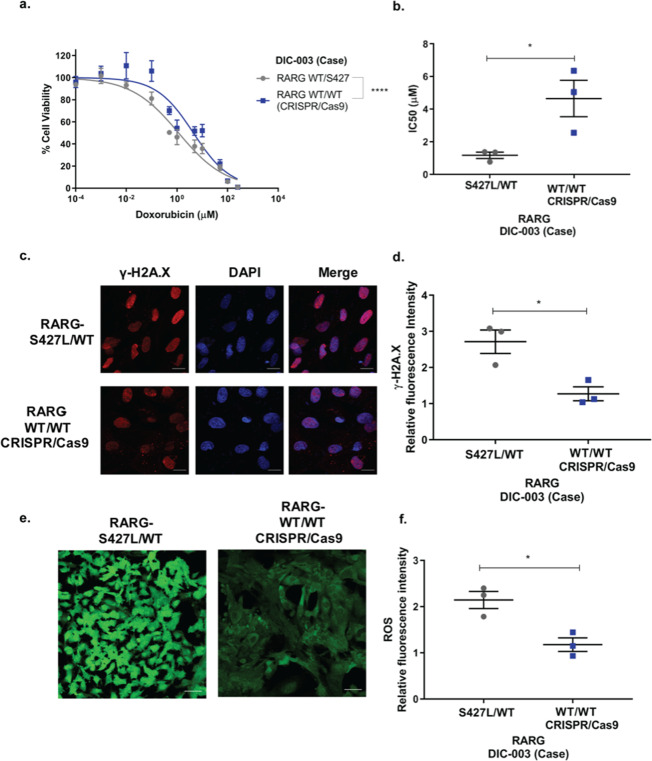
Genetic correction of *RARG*-S427L in iPSC-CMs decreases sensitivity to doxorubicin-induced cardiotoxicity. (**a**) Cell viability curve and (**b**) IC_50_ of *RARG*-S427L/WT and its corrected isogenic control (WT/WT) assessed using Cell Titer-Glo after 24 h of doxorubicin incubation (n = 3). (**c**) Representative images showing staining for γH2A.X. Scale bar = 20 μm. (**d**) Relative to vehicle control fluorescent intensity of γH2A.X staining (n = 3). (**e**) Representative images showing reactive oxygen species (ROS) production, scale bar = 50 μm. (**f**) Relative to vehicle control fluorescent intensity of ROS production (n = 3). All experiments represent biological, independent replicates, each experiment was conducted in triplicates and are shown as mean ± s.e.m., *p < 0.05, ****p < 0.0001, t-test.

To assess the functional effect of *RARG*-S427L, we performed assays of cell viability in genome-edited iPSC-CMs and their isogenic controls. Genetic correction of S427L to wild type in iPSC-CMs from a DIC case (DIC-003, Table 1) resulted in significantly reduced doxorubicin-induced cell death (Fig. 4a,b, IC_50_ 1.2μΜ ± 0.34 μΜ vs. 4.4Μμ ± 1.9 μΜ, p = 0.04, n = 3). We further investigated the impact of *RARG*-S427L on doxorubicin-induced DNA double strand breaks (DSBs) and reactive oxygen species (ROS) production, both validated assays of DIC in iPSC-CMs^7,8^. Correction of S427L to wild type resulted in reduced staining for γ-Η2Α.X, a marker of DSB, indicating less doxorubicin-induced DSBs, relative to iPSC-CMs heterozygous for S427L (Fig. 4c,d, 2.71 ± 0.56 vs. 1.27 ± 0.33 relative fluorescent units, p = 0.02, n = 3). Similarly, we found a statistically significant reduction in doxorubicin-induced ROS production in the genome-corrected compared to the heterozygous iPSC-CMs (Fig. 4e,f, 2.14 ± 0.32 vs. 1.18 ± 0.26 relative fluorescent units, p = 0.02, n = 3). These findings establish that the presence of *RARG*-S427L increases susceptibility to these important cellular phenotypes of DIC.

**Figure 5:**
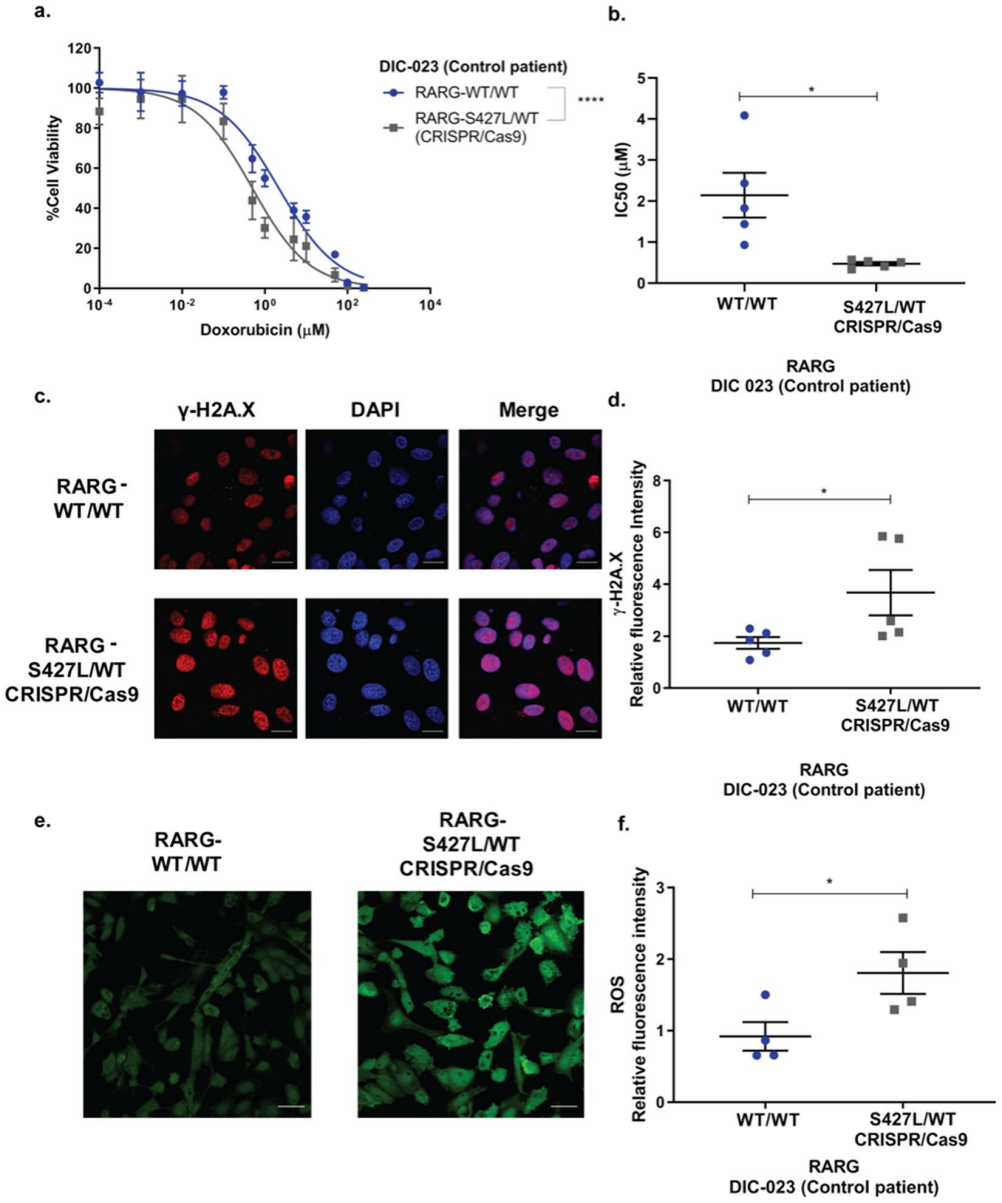
Introduction of *RARG*-S427L in iPSC-CMs increases sensitivity to doxorubicin-induced cardiotoxicity. (**a**) Cell viability curve and (**b**) IC_50_ of *RARG*-WT/WT and its isogenic cell line with genome edited S427L/WT pair (n = 5). (**c**) Representative images showing staining for γH2A.x. Scale bar = 20μm. (**d**) Relative to vehicle control fluorescent intensity of γH2A.X (n = 5). (**e**) Representative images showing ROS production. Scale bar = 50 μm. (**f**) Relative to vehicle control ROS production (n = 4). All experiments represent biological, independent replicates, each experiment was conducted in triplicates and are shown as mean ± s.e.m., *p < 0.05, ****p < 0.0001, t-test.

Next, to investigate if the S427L variant is sufficient to increase susceptibility to DIC, we introduced this variant into the iPSCs of a control individual (DIC-023, Table 1) who was wild type for *RARG* (Fig. 3a,d). Introduction of S427L resulted in a significant increase in sensitivity to doxorubicin-induced cell death (Fig. 5a,b, IC_50_. 2.14 ± 1.2 μΜ vs. 0.53 μΜ ± 0.08 μΜ, p = 0.02, n = 5), indicating that the presence of this variant is sufficient to increase susceptibility to DIC *in vitro*. Introduction of *RARG*-S427L also led to increased doxorubicin-induced DSBs (Fig. 5c,d, 1.74 ± 0.51 vs 3.67 ± 1.75 relative fluorescent units, p = 0.04 n = 5) and ROS production (Fig. 5e,f, 0.91 ± 0.40 vs 1.8 ± 0.59 relative fluorescent units, p = 0.046, n = 4).

**Figure 6:**
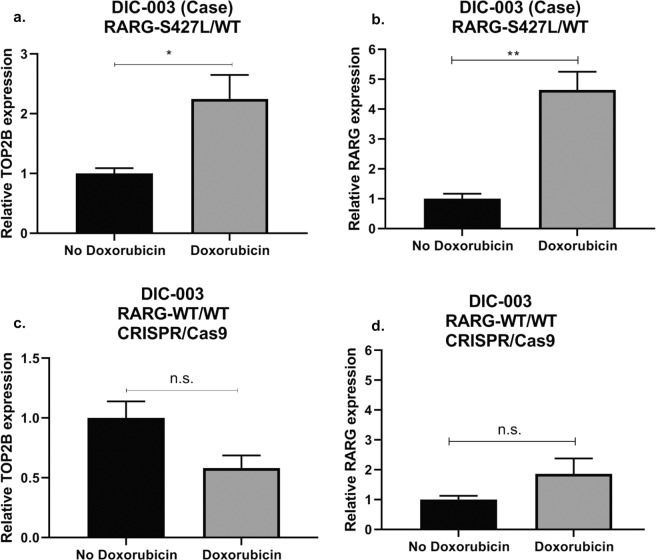
Effect of doxorubicin treatment on *TOP2B* and *RARG* in *RARG*-S427L/WT and corrected *RARG*-WT/WT. RT-qPCR gene expression of (**a**) *TOP2B* and (**b**) *RARG* relative to vehicle control in *RARG*-S427L/WT, (**c**) *TOP2B* and (**d**) *RARG* expression relative to vehicle control in corrected *RARG*-WT/WT. Data are biological, independent replicates and are shown as mean ± s.e.m., n = 3, *p < 0.05, **p < 0.01, n.s. = not significant, t-test.

A potential mechanism by which *RARG*-S427L could make cardiomyocytes more sensitive to doxorubicin is via differential regulation of topoisomerase IIβ (*TOP2B)* compared to wild type^5^. *TOP2B* is thought to be a critical mediator of DIC and RARG has been shown to bind to the promoter of *TOP2B* affecting its transcription^8,25,26^. We investigated the regulation of *TOP2B* expression by doxorubicin in *RARG*-S427L/WT iPSC-CMs and the isogenic corrected WT/WT cell line. In *RARG*-S427L/WT, *TOP2B* and *RARG* expression increased in response to doxorubicin treatment (Fig. 6a,b, Supplementary Fig. S7). In contrast, *TOP2B* expression decreased in response to doxorubicin in genetically corrected *RARG*-WT/WT iPSC-CMs (Fig. 6c) while *RARG* expression did not (Fig. 6d). In both the case with the S427L variant and in the control which the S427L variant was inserted via CRISPR/Cas9 we observed increased upregulation of *RARG* and *TOP2B* compared to their isogenic WT/WT iPSC-CMs (Supplementary Fig. S7a–d). This suggests that *RARG*-S427L increases susceptibility to DIC, in part, by leading to de-repression of *TOP2B*.

**Figure 7:**
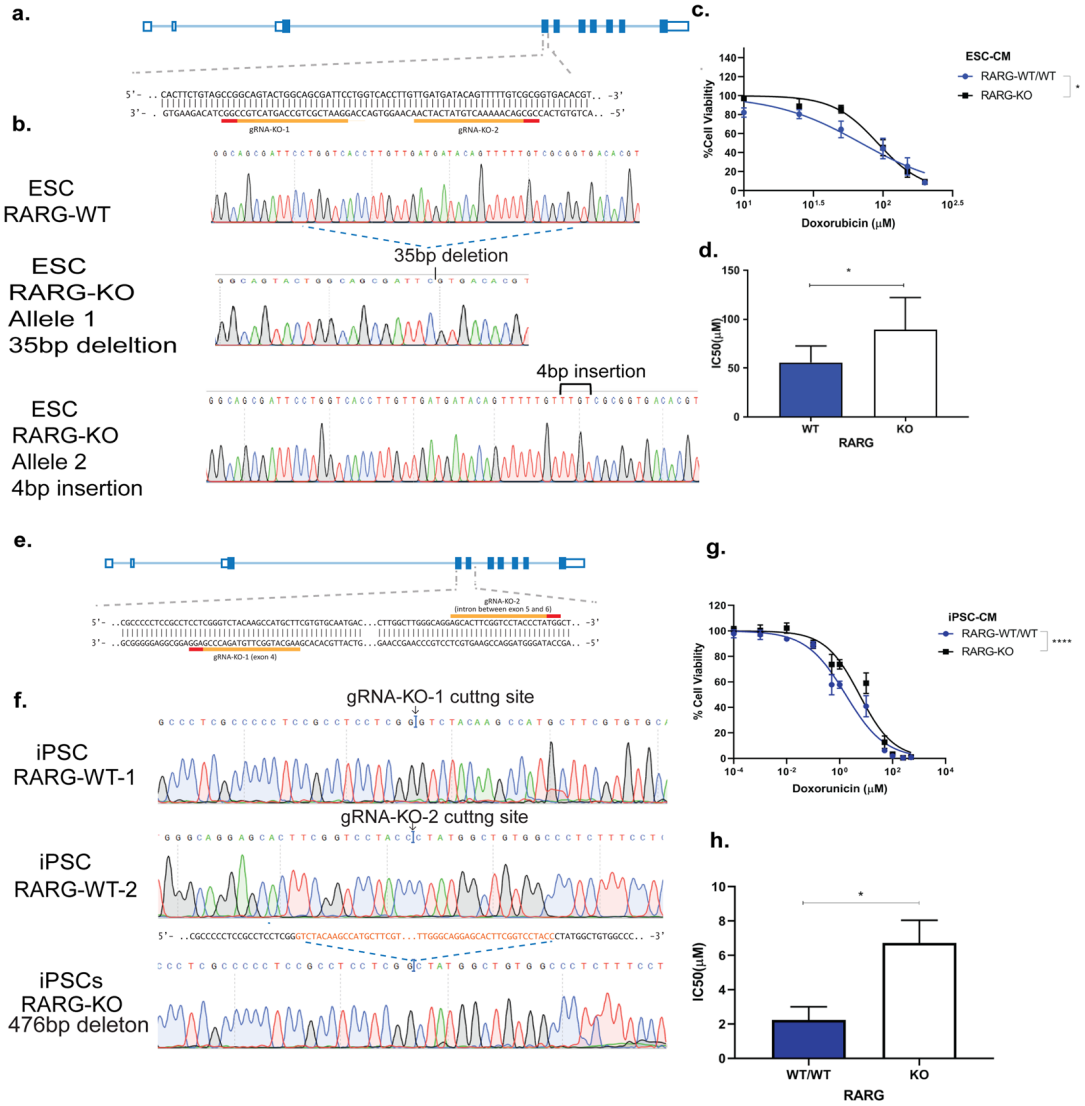
Disruption of RARG protects from doxorubicin induced cardiotoxicity. (**a**) Schematic overview of *RARG* locus and CRISPR/Cas9 knock out in ESC-CMs. (**b**) RARG-KO Sanger sequencing results in ESCs, showing a 35 base pair (bp) deletion in one allele and a 4 bp insertion in the other allele. (**c**) Cell viability curve and (**d**) IC_50_ of *RARG*-WT/WT and its isogenic cell line with disruption of *RARG* gene in ESC-CMs (n = 6). (**e**) Schematic overview of *RARG* locus and CRISPR/Cas9 knock out in iPSC-CMs from a control patient. (**f**) RARG-KO Sanger sequencing results in iPSCs, showing a 476 bp deletion. (**g**) Cell viability curve and (**h**) IC_50_ of *RARG*-WT/WT cell line and KO in iPSC-CMs (n = 4). Data are biological, independent replicates shown as mean ± s.e.m, *p < 0.05, ****p < 0.0001, t-test.

Our finding of increased mRNA expression of *RARG* in iPSC-CMs that carry the S427L allele suggests that the increased expression of this gene may play a role in the increased susceptibility to DIC. As such, we hypothesized that genetic deletion of *RARG* may be protective against DIC. We used CRISPR/Cas9 to disrupt *RARG* in two different cell lines, an embryonic stem cell line (ESC) (Fig. 7a–d) and in a control patient iPSC line (DIC-014, Table 1, Fig. 7e–h). In both cell lines, genetic disruption of *RARG* led to a significant reduction in *RARG* expression (Supplementary Fig. S8a,b). Upon doxorubicin treatment, *RARG* knock out lines showed protection from DIC (Fig. 7c,d,g and h IC50: WT/WT = 69.74μΜ and KO = 91.80μΜ, p = 0.04, n = 6 in ESC-CM, IC50: WT/WT = 2.2 ± 1.54μΜ and KO = 6.7 ± 2.64μΜ p = 0.02, n = 4 in iPSC-CMs), consistent with the concept that increased *RARG* expression is associated with increased susceptibility to DIC.

## Discussion

Here we show that a genetic variant associated with DIC in GWAS^5^ has a direct and causal relationship with DIC in patient-specific iPSC-CMs. The major findings of our study are: that iPSC-CMs from individuals who have suffered from DIC retain their sensitivity to doxorubicin *in vitro*; genetic correction of the S427L variant reduces sensitivity to DIC; introduction of this variant increases the sensitivity to DIC; and, that disruption of *RARG* protects from DIC.

Genetic association studies can identify genomic loci associated with specific traits, but cannot prove causality of a specific genetic variant, because of the possibility of linkage disequilibrium with other observed or unobserved genetic variants. While *RARG*-S427L has been implicated in DIC through GWAS, the results have been inconsistent, and the directionality of effect has been unclear^5,6^. Our results provide evidence that S427L directly increases sensitivity to DIC, independent of other genetic variants. An important strength of our study is that we were able to assess the functional impact of S427L in its endogenous genomic context, and without other changes to the genome, proving that this variant itself plays a functional role in increasing susceptibility to DIC. This result has important implications for the use of pharmacogenetic testing for this variant to identify individuals who may be at high risk for DIC.

Our finding that iPSC-CMs from DIC cases are more sensitive to doxorubicin than iPSC-CMs from doxorubicin-treated controls provides important independent validation of a previous finding^7^ and further supports the notion that iPSC-CMs represent a useful model system for dissecting the molecular mechanisms of DIC^8^ or other forms of drug-induced cardiotoxicity. An important implication of this finding is that patient-specific iPSC-CMs may be able to predict an individual’s response to a medication prior to their treatment with it. While this concept would require further validation in a prospective manner, this raises the intriguing possibility that iPSC-CMs could serve as a useful model system, in general, for assessing individual response to potentially cardiotoxic medications.

Our data have implications for understanding the mechanism of how *RARG* influences risk of DIC. Our finding that the presence of the S427L allele leads to increased expression of *RARG* and increased susceptibility to DIC suggests that *RARG* is associated with increased cell death. Similarly, we found that disruption of *RARG* decreased susceptibility to DIC. This is consistent with a recent study which showed that knocking out *RARG* in mouse embryonic fibroblasts protects cells from chemotherapeutic DNA damaging agents including doxorubicin^27^. We also found that *RARG*-S427L leads to doxorubicin-induced upregulation of *TOP2B*, which is a key player in the pathogenesis of DIC as doxorubicin can stabilize the interaction of *TOP2B* with the DNA and prevent its re-ligation, generating double strand breaks^25^. *RARG* is a transcription factor that can both repress and activate gene expression and can bind to the promoter of *TOP2B*^26^, directly affecting its transcription. As a result, this finding could be due to impaired repression (or increased activation) of *TOP2B* by *RARG*-S427L as has been previously suggested^5^. Interestingly this is further supported by RARG protein structure as S427L is located in the F-domain of RARG, nine amino acids downstream of the ligand binding domain (LBD)^28^. The role of the F-domain is to stabilize the LBD^29^. Conformational changes of the LBD expose binding sites for co-activators and co-repressors^30,31^. In RARB, which shares 73% sequence identity with RARG, a frame shift mutation at amino acid position 426 in the F terminal domain lesds to increased co-repressor binding, while deletion of the F-domain results in increased co-activator binding in RARG^29–31^. As a result, it is possible that RARG- S427L could result in a favored activation versus repressive state leading to greater expression of *RARG* and *TOP2B*. Our finding of differential regulation of TOP2B by doxorubicin in iPSC-CMs with or without S427L suggests that RARG influences susceptibility to DIC via a mechanism that involves TOP2B.

Our study has limitations that merit consideration. First, the patients who experienced DIC are older in age than the controls and therefore possess more comorbidities. Secondly, to characterize our iPSCs we used commercially available kits for trilineage differentiation potential and karyotypic abnormalities instead of the gold-standards teratoma and karyotyping respectively. However, for the purpose of this study this approach is reasonable and in line with common practice in the field of disease modelling. Furthermore, iPSC-CMs are not a pure population of cells. Recent single cell RNA-seq data have revealed transcriptional heterogeneity in iPSC-CMs arising at different stages of the differentiation process^32–34^. In addition, iPSC-CMs are a mixture of cardiac cells, including nodal-, atrial- and ventricular-like cells, with ventricular-like cells being the most abundant. While ventricular cells appear to be the most relevant for the clinical phenotype of DIC, whether doxorubicin has cell-type specific effects is unknown and requires further study. Moreover, the *in vitro* cellular assays shown here assess primarily the acute effects of doxorubicin. While the acute toxic effects of doxorubicin are thought to contribute to the longer term cardiotoxicity of this drug^35,36^, further studies will be needed to explore the more chronic effects of this drug using the iPSC-CM model. An additional issue is that the iPSC-CMs model is largely limited to investigation of cell-autonomous effects of doxorubicin. More complex effects that may result from crosstalk between different cell types or organ systems may not be accurately recapitulated using this approach. Moreover, while our results establish a functional role of *RARG*-S427L in DIC, this variant is only carried by ~30% of individuals who experience DIC^5^, and therefore cannot account for, in entirety, the increased susceptibility to DIC observed in iPSC-CMs from cases. This implies that while *RARG*-S427L represents one important pathogenic mechanism that leads to DIC, other mechanisms are likely to exist as well. Additional characterization of these cells may lead to the identification of other genetic or epigenetic factors that modulate sensitivity to doxorubicin. Finally, this study involved one case who was a carrier of the S427L variant, in which the genotype was corrected to wild type, and one cell line from a drug-tolerant control, in which the S427L variant was introduced. The advantage of this approach is that each cell line serves as its own isogenic control, and differs only at the S427L locus, which allows us to draw conclusions about the effect of this variant in isolation of other variables. However, more subjects harboring this mutation and who experienced DIC are needed to further support these findings and strengthen our finding that *RARG*-S427L is a causal variant for DIC.

In summary, we show that iPSC-CMs can identify individuals at risk for DIC, and that *RARG*-S427L has a direct and causal role in increasing susceptibility to doxorubicin. These results have implications for personalized risk prediction to prevent this devastating adverse drug reaction.

## Methods

### Participation recruitment and subject details

Participants were recruited from the Cardio-oncology Clinic at Vancouver General Hospital and provided written informed consent. Exclusion and inclusion criteria are shown in Supplementary Table S1. In total, we generated iPSCs from 7 female subjects (4 cases and 3 controls). Details of these individuals are provided in Table 1. This study was approved by the University of British Columbia-Providence Health Care (UBC-PHC) Research Ethics Board. All experiments were performed in accordance with relevant guidelines and regulations.

### TaqMan genotyping

Saliva samples were used to extract DNA (DNAgenotek). SNP Taqman genotyping assay (Invitrogen) with the rs2229774 probe was used to genotype *RARG*-S427L.

### iPSC generation and characterization

Peripheral blood mononuclear cells (PBMCs) were isolated from patients’ blood using SepMate PBMC isolation protocol (Stemcell Technologies). PBMCs in medium containing IL-2, Fetal Bovine Serum (FBS) and RPMI 1640 were incubated in a CD-3 coated plate for 5 days, as previously described^37^. CytoTune Sendai iPSC reprogramming Kit (Life Technologies) was used to deliver the Yamanaka factors in 500,000 T-activated cells. After 24 hours of infection, cells were collected and seeded in plates coated with mouse embryonic fibroblasts in Primate ES Cell media (Reprocell) supplemented with basic fibroblast growth factor (b-fgf). Cells were allowed to grow for 2–3 weeks (changing media every 2 days) and emerging colonies were picked manually and seeded in Matrigel coated plates and were cultured in mTeSR1 (Stemcell Technologies). Subsequent passaging was performed when confluency was reached (5–7 days). Versene 0.02% EDTA (Life Technologies) was used as a dissociating reagent, and mTeSR1 with Rock inhibitor (Y27632, Tocris Bioscience) at 10 μM concentration was used for day 1 of passaging and then mTeSR1 was changed daily. Cultures were kept at 37 °C and 5% CO_2_ incubators. Pluripotency characterization was performed using RT-qPCR for OCT4 and NANOG and protein expression using confocal microscopy with Tra 1–60 and NANOG antibodies. hPSC Analysis Kit (Stemcell Technologies) was used to detect karyotypic abnormalities following the manufacturer’s protocol. Trilineage differentiation was performed using the StemDiff Trilineage Differentiation Kit (Stemcell Technologies) following the manufacturer’s protocol, and differentiation was characterized using RT-qPCR with markers for endoderm (SOX17, FOXA2), mesoderm (Brachyury (T), MIXL1, CDX2) and ectoderm (OTX2, PAX 6).

### iPSCs characterization with confocal microscopy

iPSCs were harvested using Versene 0.02% EDTA (Life Technologies) and were seeded on Matrigel coated coverslips and were let to grow for 5 days in mTeSR1. Cells were washed with 3 X PBS, fixed using 4% parafomaldehyde (15 mins), washed and then permabilized with 0.2% Triton-X (15 mins), washed and blocked for 3 hours in 3% goat serum and incubated with Tra 1–60 (Abcam) and NANOG (Abcam) overnight in 4 °C. Cells were washed with 3 X PBS and incubated for 1 hour with secondary antibodies Alexa-Fluor 488 (Abcam) and Alexa-Fluor 563 (Abcam). Cells were washed with 3 X PBS and mounted on a microscope slide with antifade medium containing DAPI (VECTASHIELD, VECTOR laboratories).

### Quantitative real time PCR

After RNA extraction (RNeasy mini kit, Qiagen) and cDNA synthesis (SuperscriptR II Reverse Transcription Kit, Life Technologies), quantitative real-time PCR was performed on Viia 7 Real Time PCR System (Thermo Fisher Scientific) using SYBR Select Master Mix (Life Technologies) with a volume of 10 μL. The concentration of mRNA was normalized to the mRNA of β-actin, and fold change was calculated using the ΔΔ_Ct_. Data on graphs represent biological, independent replicates conducted at least three independent times, with each experiment conducted in technical triplicates.

### CRISPR/Cas9 genome editing in iPSCs

CRISPR/Cas9 techniques were adapted from published protocols^38–44^. To knock out *RARG*, we designed a pair of 20 bp guide RNAs targeting exon 4 and the intron between exon 5 and 6 of the *RARG* gene using GPP sgRNA Designer (https://portals.broadinstitute.org/gpp/public/analysis-tools/sgrna-design)^43,44^. This created a 476 bp deletion in the genome, a 76 amino acid deletion on the DNA binding domain of RARG protein and a premature stop codon was introduced in exon 6. To edit the *RARG* gene in the variant site (to correct or introduce the variant) we designed a 20 bp gRNA (gRNA-HDR) targeting exon 10 (using GPP sgRNA Designer) and two 127 nt single-stranded oligodeoxynucleotides (ssODNs) homology templates with or without the S427L variant that both had the same silencing mutation at the PAM site^38,39,44^. Electroporation (Amaxa Nucleofector 2b with program A-023) was used to deliver 5 µL of ribonucleoprotein (RNP) complex, 120 pmol *in vitro* synthesized gRNA duplex (crRNA:tracrRNA), 104 pmol Cas9 nuclease (Integrated DNA Technologies, following manufacturer’s instructions) and 2 pmol ssODNs in 100 µL of 8 × 10^5^ iPSCs using Human Stem Cell Nucleofector Kit 1(Lonza, following manufacturer’s instructions)^40,42^. Subsequently, cells were seeded on Matrigel-coated 6-well plates (for 48 hours) and then seeded in 10 cm dishes at a low density (for 9 days)^41^. Colonies were then picked manually, transferred to a 96 well plate and when confluency was reached genomic DNA was extracted by QuickExtract DNA Extraction Solution (Epicentre)^38,45^. To identify colonies of interest Taq 5X Master Mix (New England Biolabs) was used to amplify the target region and restriction enzyme BseRI (New England Biolabs) which covers the SNP site was used to perform restriction fragment length polymorphism (RFLP) assay^38^. Colonies with targeted 476 bp deletion for the *RARG*-KO were identified based on different DNA fragment size of PCR products that separated on agarose gels. The PCR products of colonies of interest were purified by QIAquick PCR Purification Kit (QIAGEN) and sequenced by Sanger sequencing. (More details on this method are in Supplemental Information).

### CRISPR/Cas9 genome editing in ESCs

CRISPR/Cas9 techniques were adapted from published protocols^8,38,46^. A pair of single guide RNAs targeting exon 5 of the *RARG* gene were cloned into the BbsI site of pSpCas9n(BB)-2A-Puro (PX462) (Addgene). Neon Transfection System (Life Technologies) was used to insert 2.5 μg of plasmid DNA into 1 × 10^6^ NKX2-5^eGFP/w^ cells. After electroporation the cells were seeded on a Matrigel-coated 6 well plates and twenty four hours later they were treated with 1 μg/mL puromycin for 24 h. Two weeks later colonies were picked manually and genomic DNA was extracted in order to PCR amplify the *RARG* gene. T7E1 was used to identify mutations which was digested and assessed by gel electrophoresis. The purified PCR products of positive clones were cloned in pGem-T easy vector (Promega) and transformed in One Shot TOP10 E.coli competent cells (Life Technologies). After ampicillin selection and amplification, DNA from minimum 10 bacterial clones per sample was extracted and sent for Sanger sequencing. The resulting clone had a 35pb deletion in one allele and a 4pb insertion in the other, creating a premature stop codon on exon 6.

### iPSC-CM differentiation and characterization

iPSCs were differentiated to CMs using Wnt activation with CHIR99021 (day 0) and Wnt inhibition with IWP2 (day 3) cultured in RPMI and B27 medium without insulin. At day 5 medium was changed to RPMI/B27 without insulin and at day 7 onwards RPMI/B27 with insulin was used^47^. Spontaneous beating was observed on day 8. iPSC-CMs were characterized using qPCR and flow cytometry for cTnT and MYH7. For each experiment, we performed biological replicates using different differentiation batches of the same iPSC clone. All experiments were performed between days 30–45 of differentiation.

### Flow cytometry

iPSC-CMs were dissociated using 0.5U/ml Liberase (Roche), >50 U/ml DnaseI (Stemcell Technologies) in RPMI medium and then trypsinized. Cells were washed with DPBS, stained with live-dead stain (Fixable Viability Stain 520, BD Horizon) and anti-cTnT-BV421 antibodies (BD Horizon). Dead cells were excluded during flow cytometry analysis and gating was determined using an isotype antibody. Flow cytometry data were acquired on Gallios Flow Cytometer and analyzed on Kaluza.

### iPSC-CMs characterization with confocal microscopy

Cells were seeded on Matrigel-coated coverslips and were left to attach for 2 days. Cells were washed with 3 X PBS, fixed with 4% parafomaldehyde (15 mins), washed and permabilized with 0.2% Triton-X (15 mins), washed and blocked for 3 hours in 3% goat serum and incubated with cTnT (Abcam) and sarcomeric α-actinin (Abcam) overnight. Cells were washed with 3 X PBS and incubated for one hour with secondary antibody Alexa Fluor-563 (Abcam) and Alexa Fluor-488 (Abcam) and were then washed and mounted on a slide using Antifate mounting medium containing DAPI (VECTASHIELD, VECTOR laboratories). Slides were viewed on Zeiss LSM880 confocal microscope.

### Cell viability assay

Cells were dissociated using TrypLE, seeded on 96-well Matrigel coated plates and incubated with increasing doses of doxorubicin for 24 hours. Cells were then washed with 1xPBS and incubated with Cell Titer-Glo reagents (Promega) for 10 minutes at room temperature. Luminescence was measure in Spectramax plate reader (Molecular Diagnostics). Data on graphs represent biological, independent replicates conducted at least three independent times, with each experiment conducted in technical triplicates. All experiments shown were conducted in multiple differentiation batches, from one cell line from each patient.

### Optical mapping

iPSCs-CMs were superfused with IMDM supplemented with NaCl (final concentrations in mmol/L: 140 NaCl, 3.6 KCL, 1.2 CaCl2, 1 MgCl2. 10 HEPES and 5.5 D-glucose) and loaded with 15 μM of the potentiometric voltage sensitive dye RH-237 (Molecular Probes, Eugene, OR). Blebbistatin, a myosin ATPase inhibitor (Sigma-Aldrich), was employed to avoid motion artifact. The iPSC-CMs were kept in a 37 °C plate and were excited by 532 nm LEDs. Voltage sensitive dye, RH-237 emission was monitored using >710 nm long-pass and signal was captured with a single Hamamatsu ORCA Flash 4 digital CMOS camera. Doxorubicin was diluted in DMSO and added to the culture dish in sequentially increasing concentrations (1.0, and 10.0 μM) at 20-minute intervals. Electrical field stimulation was applied using stainless steel electrodes. The electrodes were ~1 cm apart and were placed in the imaging chamber. Signal processing of the optically mapped data was performed using a custom IDL software program^23,24^. APD_50_ data represent independent, biological replicates with n = 3 cases and n = 3 controls.

### γ-Η2Α.X staining

Cells were seeded on Gelatin-coated coverslips and treated with doxorubicin for 20 hours. After treatment, cells were washed with 3 X PBS, fixed with 4% parafomaldehyde (15 mins), washed permabilized with 0.2% Triton-X (15 mins), washed and blocked for 3 hours in 3% goat serum and incubated with γ-Η2Α.X (Abcam) overnight. Cells were washed with 3 X PBS and incubated for one hour in secondary antibody Alexa Fluor-488 (Abcam) and were then washed and mounted on a slide using Antifate mounting medium containing DAPI (VECTASHIELD, VECTOR laboratories). Coverslips were viewed on Zeiss LSM880 confocal microscope. A minimum of three images were captured from each sample and were quantified using ImageJ based on fluorescence intensity of γ-Η2Α.X staining per nucleus. Graphs show intensity relative to vehicle-only treated cells with a minimum of three independent biological replicates.

### ROS production

Cells were seeded on gelatin-coated glass bottom dishes and treated with doxorubicin for 3 hours two days after seeding. After treatment, cells were washed twice with PBS and incubated 15 min at 37 °C in plain RPMI with 5 μM H2DCFDA (Thermo Fisher Scientific). Cells were washed and analyzed on Zeiss LSM880 confocal microscope. A minimum of three images were captured from each sample and were quantified based on fluorescence intensity that was measured using the ZEN blue Analysis software of the microscope. Graphs show intensity relative to vehicle-only treated cells with a minimum of three independent biological replicates.

### Statistical analysis

All statistical analyses were conducted with GraphPad Prism 5.0, 7.03 and 8.3.0 versions. In graphs, values are expressed as mean ± standard error of mean (s.e.m.) and represent independent, biological repeats (at least three) unless otherwise stated. Statistical analyses were performed using Student’s t-test when comparing two groups. Cell viability curves were also described using F-test. Signal processing of the optically mapped data was performed using a custom IDL software program^23,24^.

## Supplementary information

Supplementary Information.
