# Supplementary material for: Variation in RARG increases susceptibility to doxorubicin-induced cardiotoxicity in patient specific induced pluripotent stem cell-derived cardiomyocytes
Source: Sci Rep. 2020 Jun 25;10:10363. doi: 10.1038/s41598-020-65979-x (PMC7316788; doi:10.1038/s41598-020-65979-x)
Supplement: Supplementary file 1 — Supplementary Information. [file 41598_2020_65979_MOESM1_ESM.pdf]

Supplementary Information

Variation in *RARG* increases susceptibility to doxorubicin-induced cardiotoxicity in patient specific induced pluripotent stem cell-derived cardiomyocytes

Christidi, E.[1], Huang H.[1*], Shafaattalab S.[2,6*], Maillet A.[3], Lin E.[2], Huang K.[1], Laksman Z.[4], Davis M.K.[5], Tibbits G.F.[2,6], Brunham L.R.[1,5,7]

[1]Centre for Heart Lung Innovation, Department of Medicine, University of British Columbia, Vancouver, Canada

[2]Molecular Cardiac Physiology Group, Department of Biomedical Physiology and Kinesiology, Simon Fraser University, Burnaby, Canada

[3]Astellas Pharma Europe B.V., Leiden, Netherlands

[4]Heart Rhythm Services, Division of Cardiology, Department of Medicine, University of British Columbia, Vancouver, Canada

[5]Department of Medicine, University of British Columbia, Vancouver, British Columbia, Canada

[6]Department of Cardiovascular Science, British Columbia Children's Hospital, Vancouver, Canada

[7]Department of Medical Genetics, University of British Columbia, Vancouver, British Columbia, Canada

a.

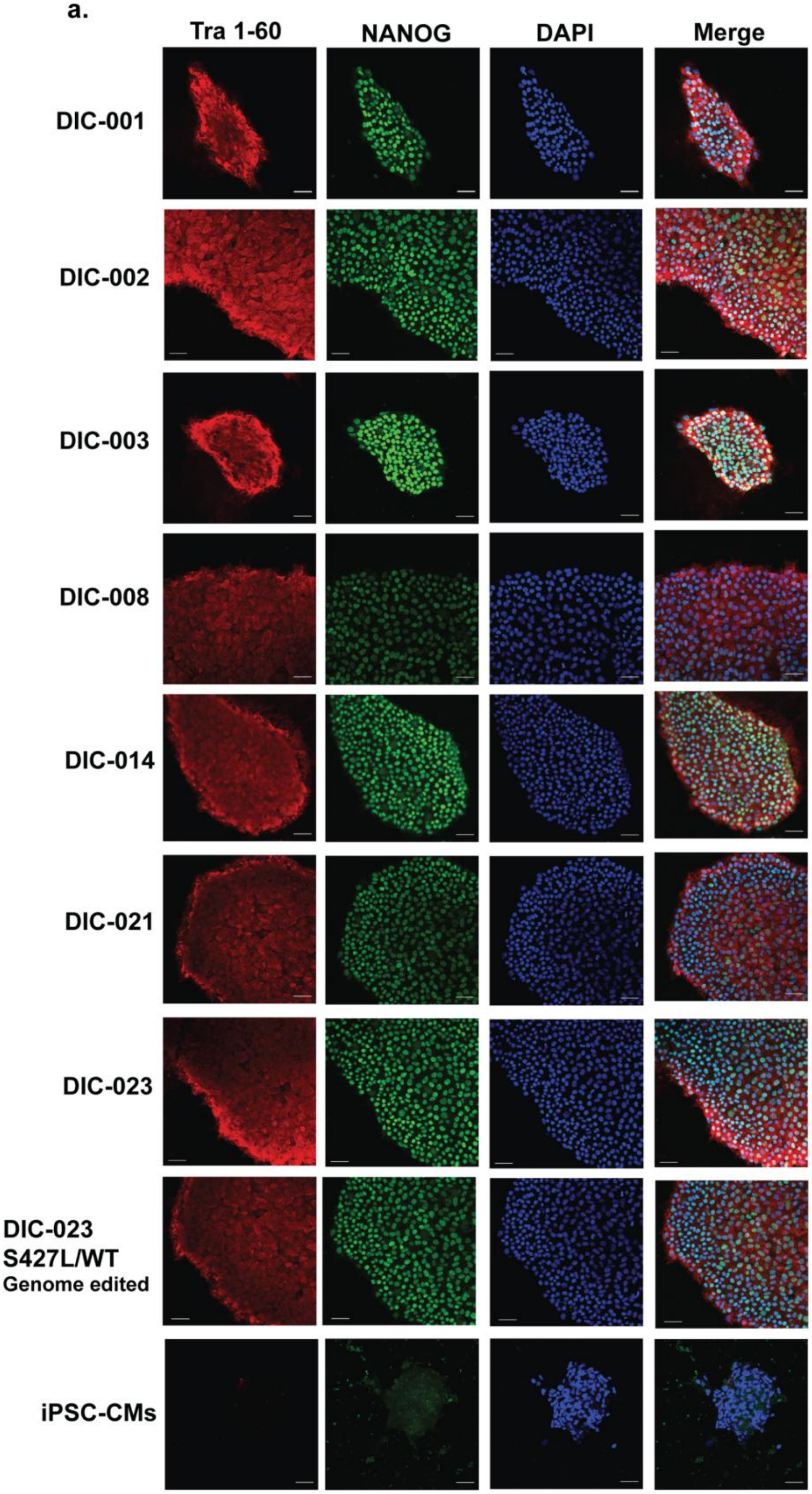
Tra 1-60
NANOG
DAPI
Merge
DIC-001
DIC-002
DIC-003
DIC-008
DIC-014
DIC-021
DIC-023
DIC-023
S427L/WT
Genome edited
iPSC-CMs

b.

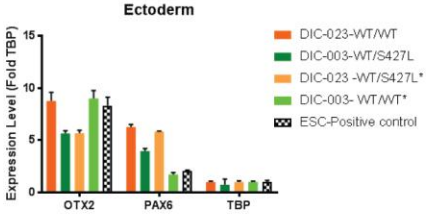
Ectoderm
Expression Level (Fold TBP)
15
10
5
0
OTX2
PAX6
TBP
DIC-023-WT/WT
DIC-003-WT/S427L
DIC-023 -WT/S427L*
DIC-003- WT/WT*
ESC-Positive control

c.

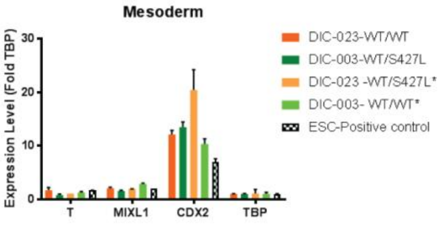
Mesoderm
Expression Level (Fold TBP)
30
20
10
0
T
MIXL1
CDX2
TBP
DIC-023-WT/WT
DIC-003-WT/S427L
DIC-023 -WT/S427L*
DIC-003- WT/WT*
ESC-Positive control

d.

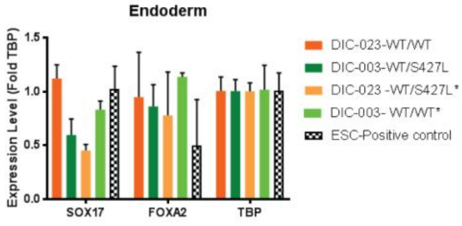
Endoderm
Expression Level (Fold TBP)
1.5
1.0
0.5
0.0
SOX17
FOXA2
TBP
DIC-023-WT/WT
DIC-003-WT/S427L
DIC-023 -WT/S427L*
DIC-003- WT/WT*
ESC-Positive control

Figure S1: Characterization of iPSCs. a) Cell lines were assessed for protein expression of pluripotency markers; Tra1-60 and NANOG. Scale Bar=20µm. b., c., d.) Cells were differentiated to; (b) ectoderm, (c.) mesoderm and (d.) endoderm using the commercially available kit from Stem cell Technologies (StemDiff Trilineage differentiation kit). RT- qPCR for ectoderm markers (OTX2, PAX6), mesoderm markers (T (Brachyury), MIXL1, CDX2) and endoderm markers (SOX17, FOXA2) were compared to a well characterized ESC cell line. TBP: TATA-Box Binding Protein, * shows genome edited cell lines.

a.

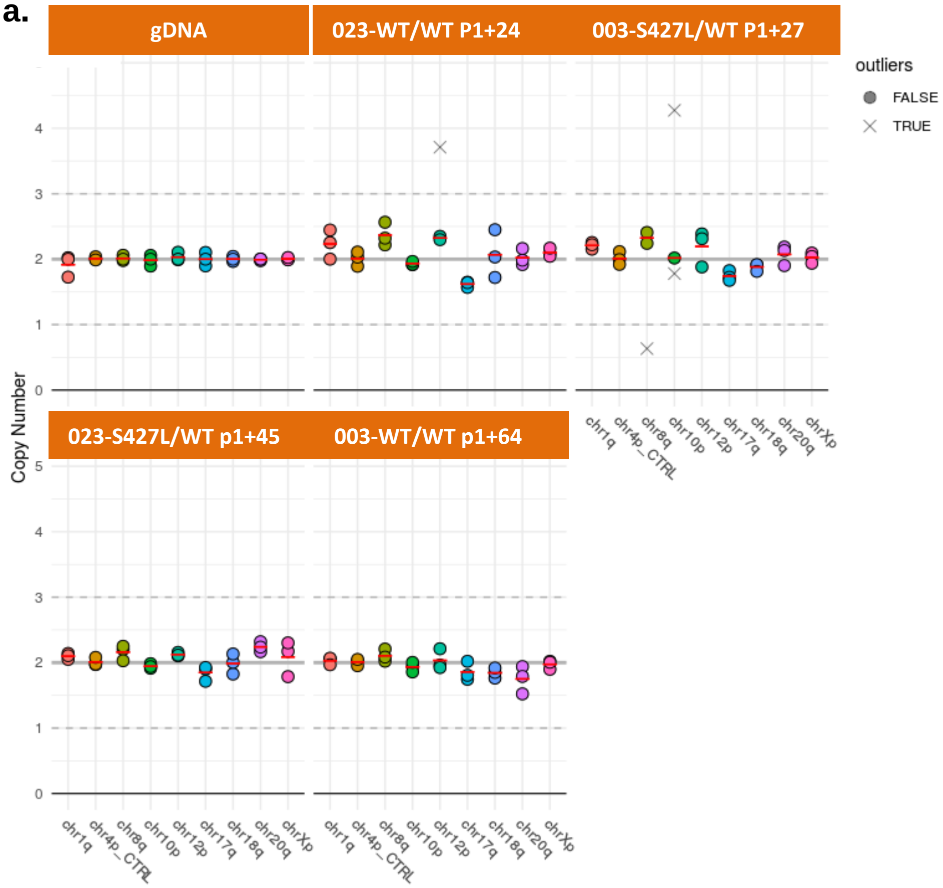
gDNA
023-WT/WT P1+24
003-S427L/WT P1+27
023-S427L/WT p1+45
003-WT/WT p1+64
outliers
FALSE
TRUE
Copy Number
0
1
2
3
4
5
chr1q
chr4p_CTRL
chr8q
chr10p
chr12p
chr17q
chr18q
chr20q
chrXp

b.

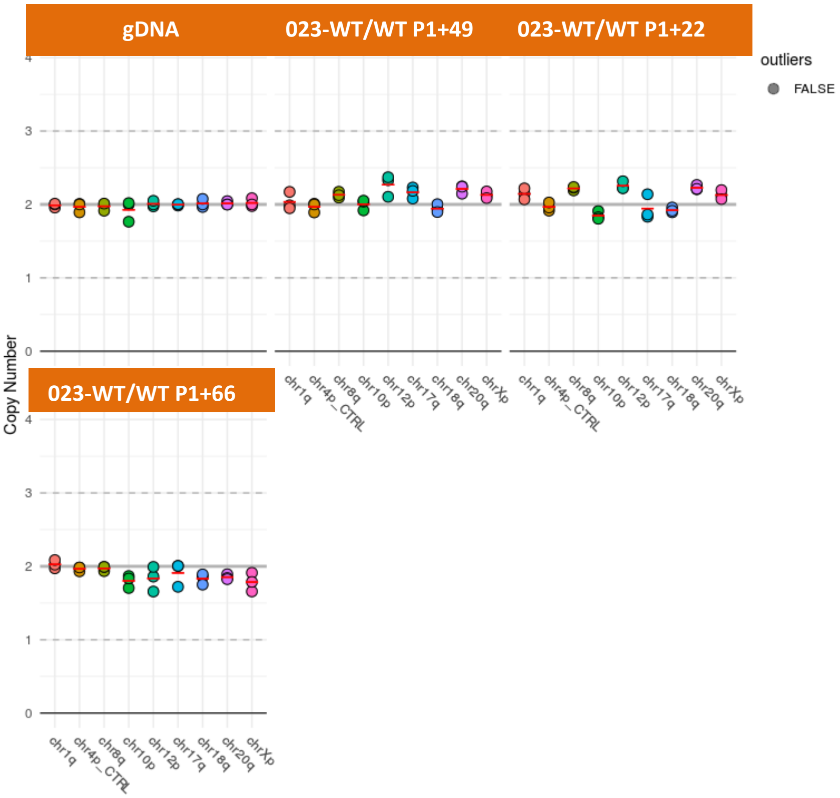
gDNA
023-WT/WT P1+49
023-WT/WT P1+22
023-WT/WT P1+66
outliers
FALSE
Copy Number
0
1
2
3
4
chr1q
chr4p_CTRL
chr8q
chr10p
chr12p
chr17q
chr18q
chr20q
chrXp

Figure S2: hPSC Genetic Analysis for the investigation of karyotypic abnormalities.

Digital karyotyping using hPSC genetic Analysis Kit (Stemcell Technologies) showing a.) normal karyotype detected in all cell lines except 023 which was flagged as "possibly abnormal " in chromosomal arm 12p. b.) Further investigation of 023 cell line shows normal karyotype except passage 49 with possible chromosomal arm 12p duplication.

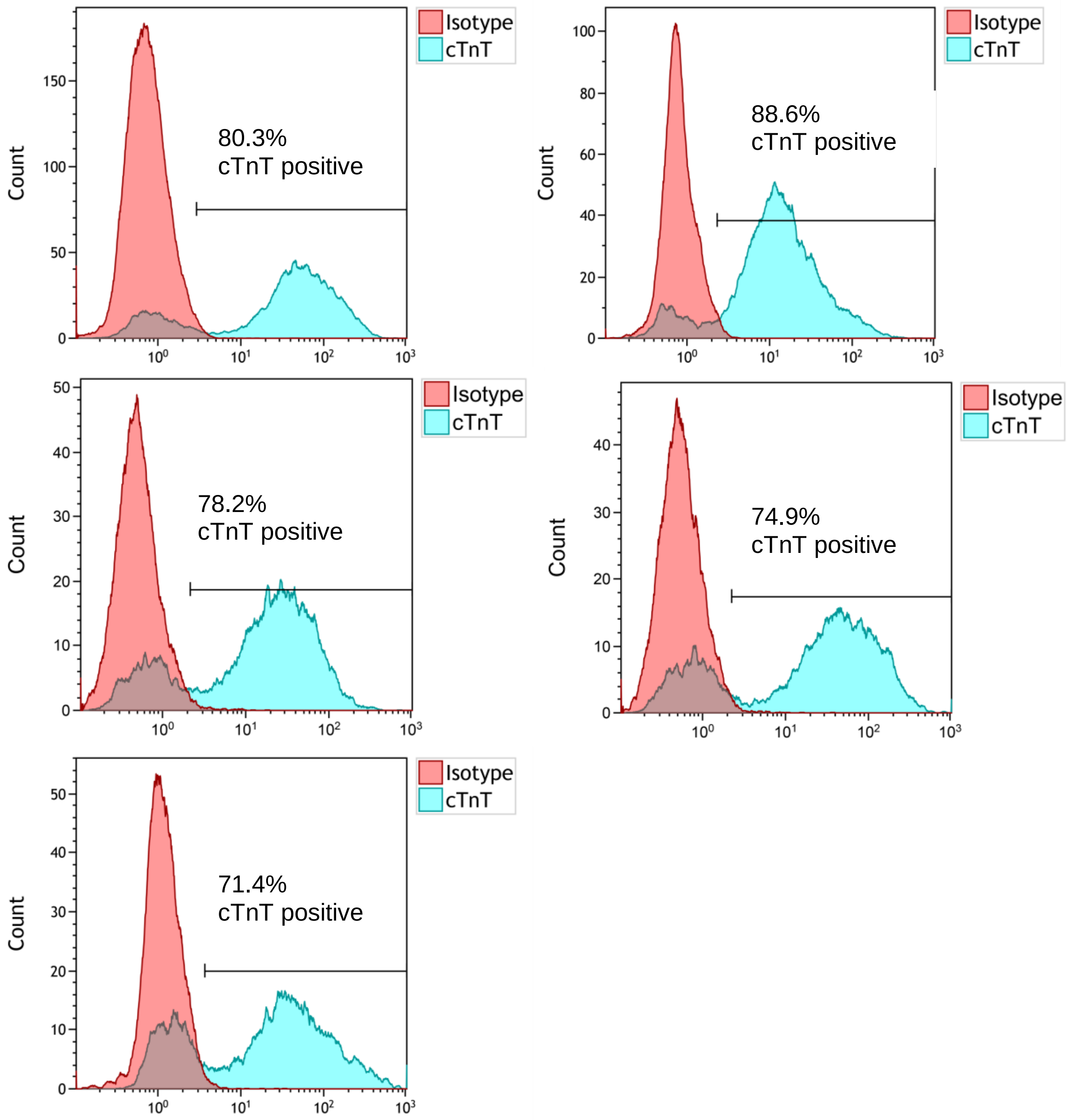

Figure S3: cTnT expression in different differentiation batches.

cTnT expression between different differentiation batches and passages of cell line DIC-023.

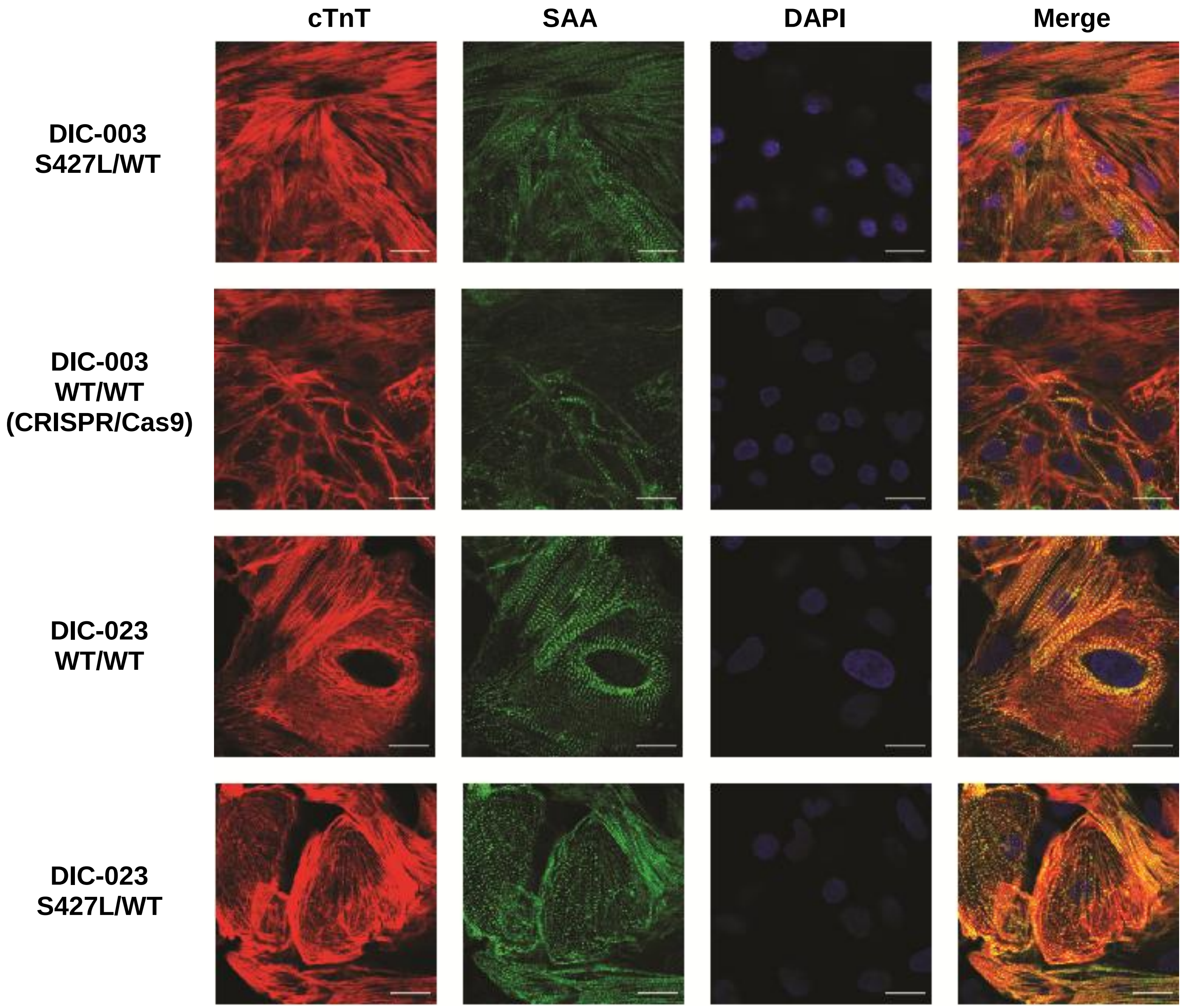

Figure S4: Cardiac Troponin T (cTnT) and sarcomeric α-actinin expression.

Confocal microscopy images showing presence of cTnT and Sarcomeric α-actinin (SAA) in different cell lines. Scale bar=20μm

A.

Cell line	Total screened colonies	Edited	HDR editing efficiency
Case S427L/WT→WT/WT	484	3	0.62%
Control WT/WT→ S427L/WT	352	6	1.70%

B.

Site	Sequence	PAM	#MM	Gene	Locus
On-target	CTCATCCTCGCTAGAGGCAT	TGG	0	RARG	chr12:+53211720
OFF-1	CTCAACCTCTCCAGAGCCAT	TGG	4	PPM1G	chr2:-27383367
OFF-2	GTCATCCTCCCCATAGGCAT	CAG	4	SAP30BP	chr17:-75668539
OFF-3	CTCTTCCTCACTGGAGTCAT	CAG	4	NOLC1	chr10:-102160919
OFF-4	CTCATCCACTGTGGAGGCAT	CAG	4	ALPK1	chr4:-112432086
OFF-5	CTCATGCTCGATAGCAGGCAT	GGG	3	LRRC4C	chr11:+40114513

C.

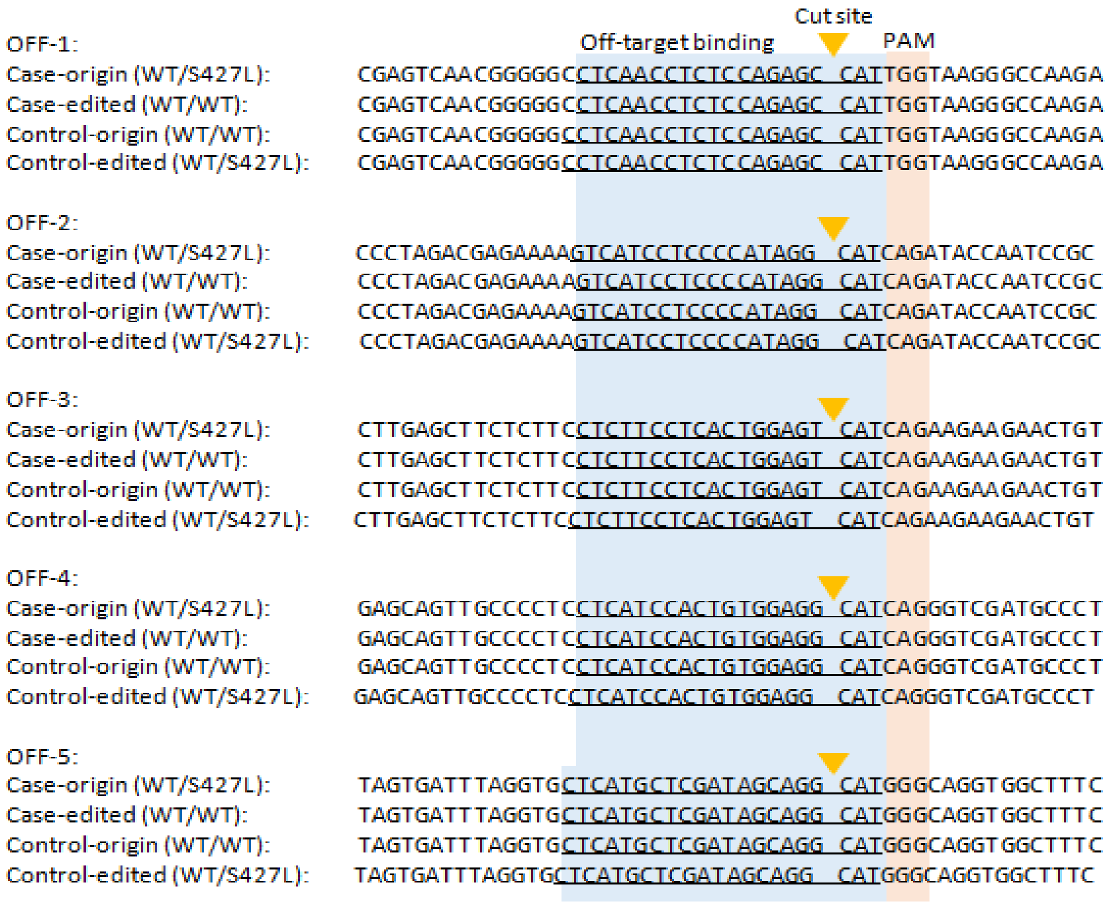

Figure S5: HDR editing efficiency and off-target validation.

a) Summary of on-target editing efficiency in case-S427L/WT (corrected to WT/WT) and control (inserted S427L). The total screened colonies refer to manually picked and screened colonies by RFLP assay. Targeted editing refers to colonies that went through HDR editing without any random insertion or deletion by NHEJ. b) Summary of on-target gRNA sequence and the top 5 *in silico* predicted potential off-target sites located at a protein coding region. Nucleotides that highlighted in red are mismatch sites compared to gRNA sequence. Mismatch number (#MM), gene symbol and sequence locus are also showed in the table. c) Sanger sequencing results of predicted potential off-target sites comparing original and edited cell lines. Regions highlighted in blue are off-target binding sites and PAM sites are in orange. Yellow arrows point at the cut sites of Cas9 nuclease.

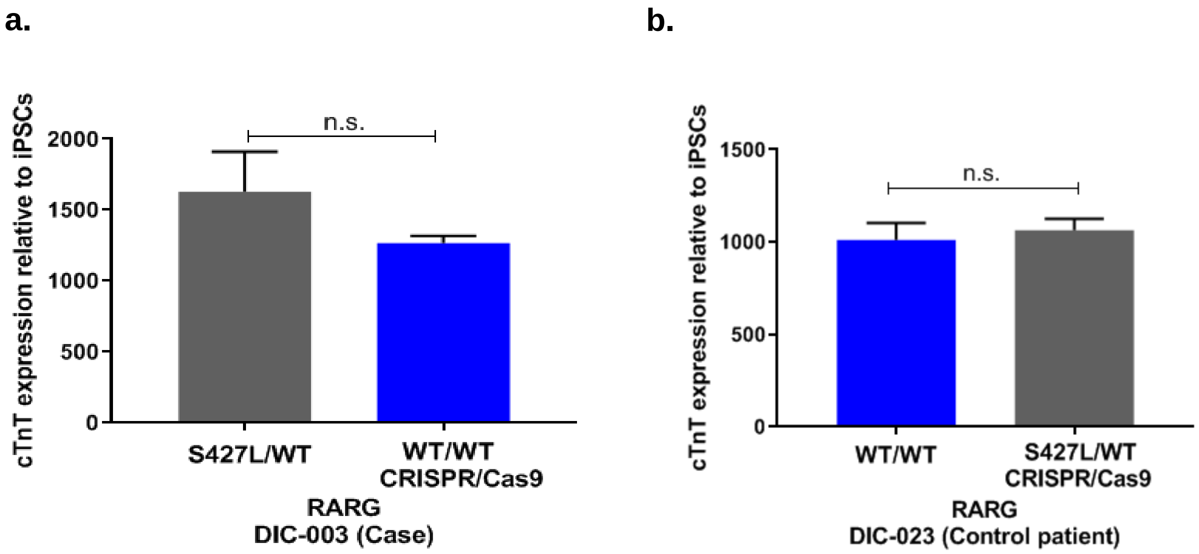

Figure S6: cTnT expression in iPSC-CMs derived from wildtype and genome edited clones.

RT-qPCR analysis for cTnT expression in a) a case (DIC-003) with the S427L/WT and the its edited WT/WT isogenic pair, b) (DIC-023) control WT/WT cell line and its isogenic genome edited with S427L/WT inserted. n=3, biological replicates, ± s.e.m, n.s.= no significance, t-test.

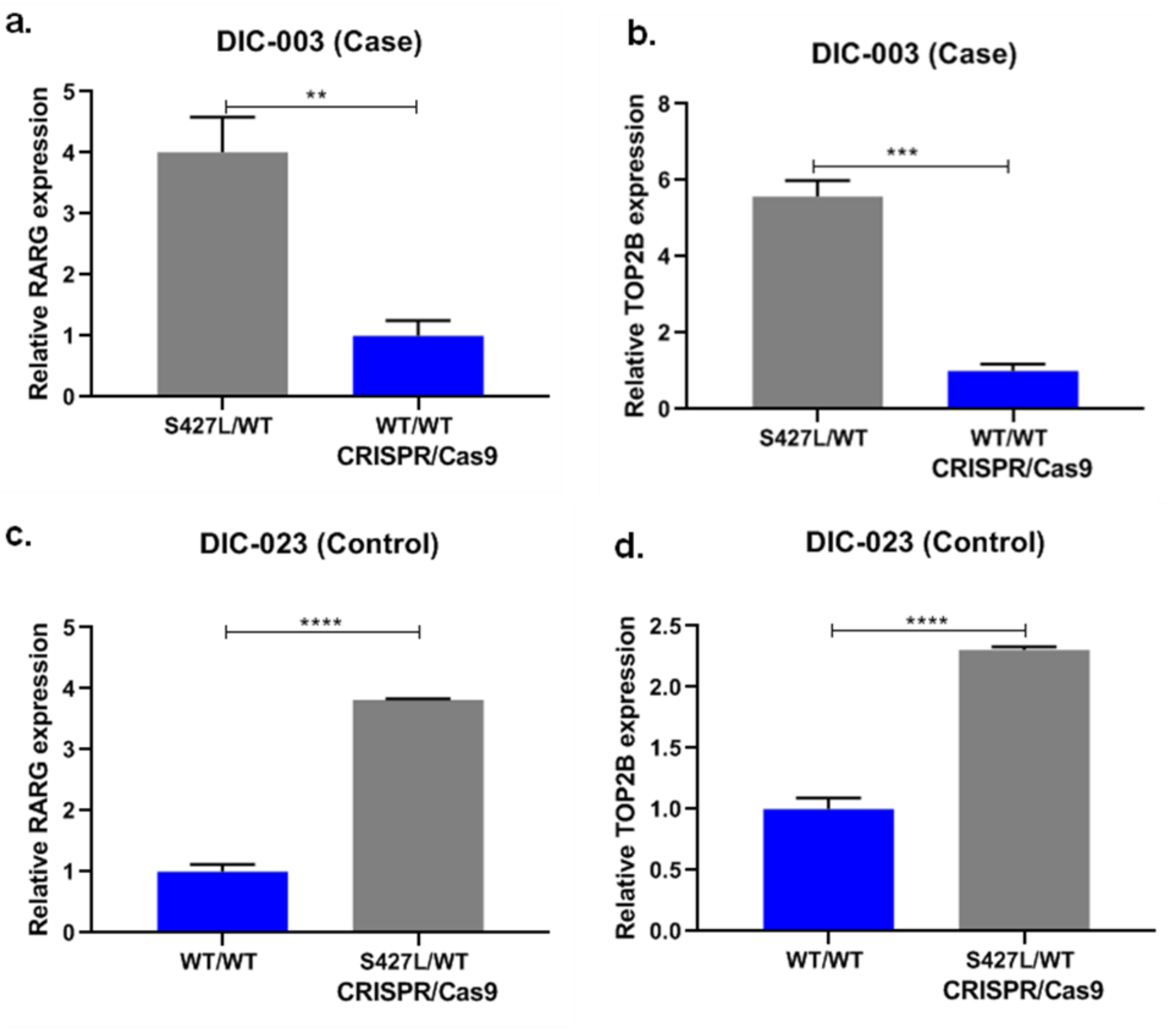

Figure S7: Comparison of *RARG* and *TOP2B* expression with doxorubicin in *RARG*-WT/WT and isogenic *RARG*-S427L/WT.

(a.) *RARG* and (b.) *TOP2B* expression in response to 1μM doxorubicin after 24 hours of treatment in iPSC-CMs from a patient carrying RARG-S427L/WT (DIC-003 case) compared to genome edited isogenic *RARG*-WT/WT. (c.) *RARG* and (d.) *TOP2B* expression in response to 1μM doxorubicin after 24 hours of treatment in iPSC-CMs from a control patient with RARG-WT/WT compared to genome edited isogenic *RARG*-S427L/WT. Expression is measured using RT-qPCR and is relative to WT/WT in all groups, n=3 biological, independent replicates, **p<0.01, ***p<0.001, ****p<0.0001, ± s.e.m, t-test.

a.

b.

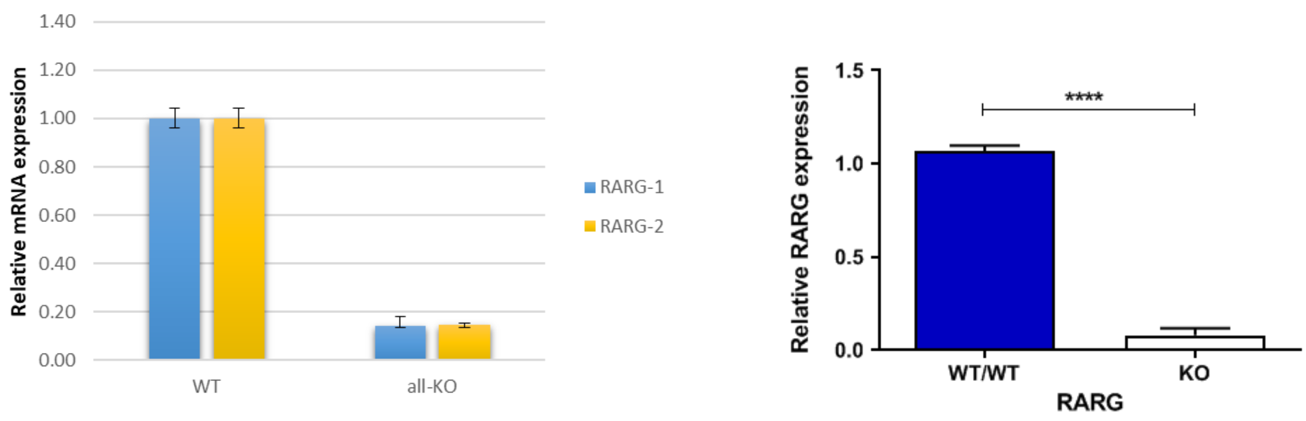

Figure S8: *RARG* expression

a)*RARG* expression of isoform 1 and 2 in iPSC-CM derived from a control patient (WT) and the CRISPR/Cas9 isogenic edited clone with disrupted RARG. b) *RARG* expression in ESC-CMs WT and CRISPR/Cas9 isogenic edited clone with disrupted RARG ****$p<0.0001$, ± s.e.m, t-test.

Table S1: Enrolment criteria.

Enrolment Criteria		
**Criteria**	**Cases**	**Control**
**Inclusion**	• Normal pre-treatment ejection fraction<br>• Recent history of Doxorubicin treatment or other anthracyclines<br>• Post treatment Ejection Fraction less than 40%	• Normal ejection fraction post treatment<br>• Administered Doxorubicin or other anthracycline 5 years ago or more
**Exclusion**	• Herceptin use<br>• History of coronary artery disease<br>• Structural, congenital or valvular cardiac disease<br>• History of alcohol consumption of > 10 drinks per week	• History of alcohol consumption of > 10 drinks per week<br>• History of heart disease

Supplemental Methods:

CRISPR/Cas9 sgRNA and templates design: A 20bp guide RNA (gRNA) was designed to target exon 10 of the RARG gene. We designed the gRNA using the Broad Institute GPP sgRNA Designer tool (https://portals.broadinstitute.org/gpp/public/analysis-tools/sgrna-design). The gRNA that was chosen had the highest score of combined on-target and off-target rank and was in the closest proximity to the variant site. HDR repairing templates, two 127nt single-stranded oligodeoxynucleotides (ssODNs) with or without the SNP were used separately for SNP insertion/correction. Design of ssODNs was done according to the published protocol[1]. Both ssODNs possess the same silencing mutation of the PAM site to avoid re-cut after successful editing.

Electroporation of ribonucleoprotein complex and colony isolation: 5 µL of ribonucleoprotein (RNP) complex together with 120 pmol of in vitro synthesized gRNA duplex (crRNA:tracrRNA), 104 pmol of Cas9 nuclease (Integrated DNA Technologies), 2 pmol ssODNs were delivered into 100 µL 8 x 10^5 iPSCs using Human Stem Cell Nucleofector Kit 1 (Lonza). Electroporation was performed using Nucleofector 2b with pre-set program A-023. After electroporation, the cells were seeded on Matrigel-coated 6-well plates, were let to recover and forty-eight hours later, the cells were seeded in low density in 10-cm dishes for isolation. CloneR (Stemcell Technologies) was used to increase the cloning efficiency. Colonies were let to grow and after 9 days they were picked manually and transferred to 96-well plates.

Screening for targeted edited colonies: Genomic DNA from the colonies was extracted using 35ul/well of QuickExtract DNA Extraction Solution (Epicentre). Taq 5X Master Mix (New England Biolabs) was used to amplify the targeted region and using the restriction enzyme BseRI (New England Biolabs) that covers the mutation site we performed restriction fragment length polymorphism (RFLP) assay to assess editing success. DNA sequences with wild-type genotype were digested and showed two smaller bands while sequences with SNP were not digested and showed one larger band. We then PCR-amplified the colonies with positive RFLP results and did Sanger sequence to validate the results (GENEWIZ). PCR products were purified by QIAquick PCR Purification Kit (QIAGEN).

CRISPR/Cas9 off-target editing evaluation: The off-target potential of gRNA was assessed by CRISPR-Cas9 guide RNA design checker (https://www.idtdna.com). The top 5 off-target hits that were located in protein coding regions were assessed. Primers for the off-target region were designed using NCBI Primer-BLAST. Taq 5X Master Mix was used to amplify off-targeted regions and PCR products were purified by QIAquick PCR Purification Kit. Purified DNA was sent for Sanger sequencing. Sequencing results were visualized using SnapGene viewer (Figure S5).

Supplementary References:

1. Richardson, C. D., Ray, G. J., DeWitt, M. A., Curie, G. L. & Corn, J. E. Enhancing homology-directed genome editing by catalytically active and inactive CRISPR-Cas9 using asymmetric donor DNA. *Nat. Biotechnol.* **34**, 339–344 (2016).
